# A Web Geographic Information System to share data and explorative analysis tools: The application to West Nile disease in the Mediterranean basin

**DOI:** 10.1371/journal.pone.0196429

**Published:** 2018-06-27

**Authors:** Lara Savini, Susanna Tora, Alessio Di Lorenzo, Daniela Cioci, Federica Monaco, Andrea Polci, Massimiliano Orsini, Paolo Calistri, Annamaria Conte

**Affiliations:** Istituto Zooprofilattico Sperimentale dell’Abruzzo e del Molise ‘G. Caporale’, Campo Boario Teramo, Italy; Oklahoma State University, UNITED STATES

## Abstract

**Background:**

In the last decades an increasing number of West Nile Disease cases was observed in equines and humans in the Mediterranean basin and surveillance systems are set up in numerous countries to manage and control the disease. The collection, storage and distribution of information on the spread of the disease becomes important for a shared intervention and control strategy. To this end, a Web Geographic Information System has been developed and disease data, climatic and environmental remote sensed data, full genome sequences of selected isolated strains are made available. This paper describes the Disease Monitoring Dashboard (DMD) web system application, the tools available for the preliminary analysis on climatic and environmental factors and the other interactive tools for epidemiological analysis.

**Methods:**

WNV occurrence data are collected from multiple official and unofficial sources. Whole genome sequences and metadata of WNV strains are retrieved from public databases or generated in the framework of the Italian surveillance activities. Climatic and environmental data are provided by NASA website. The Geographical Information System is composed by Oracle 10g Database and ESRI ArcGIS Server 10.03; the web mapping client application is developed with the ArcGIS API for Javascript and Phylocanvas library to facilitate and optimize the mash-up approach. ESRI ArcSDE 10.1 has been used to store spatial data.

**Results:**

The DMD application is accessible through a generic web browser at https://netmed.izs.it/networkMediterraneo/. The system collects data through on-line forms and automated procedures and visualizes data as interactive graphs, maps and tables. The spatial and temporal dynamic visualization of disease events is managed by a time slider that returns results on both map and epidemiological curve. Climatic and environmental data can be associated to cases through python procedures and downloaded as Excel files.

**Conclusions:**

The system compiles multiple datasets through user-friendly web tools; it integrates entomological, veterinary and human surveillance, molecular information on pathogens and environmental and climatic data. The principal result of the DMD development is the transfer and dissemination of knowledge and technologies to develop strategies for integrated prevention and control measures of animal and human diseases.

## Background

West Nile disease (WND) is one of the most widespread mosquito-borne infectious diseases in the World, caused by the West Nile virus (WNV) (Flavivirus, Flaviviridae). The transmission cycle involves wild and domestic birds as primary hosts and mosquitoes, mainly of the *Culex* genus, as vectors; humans and equines are considered dead-end hosts. WNV has been circulating in the Mediterranean basin at least since the 1960s, but no large outbreak of WNV infection was reported until the 1996 epidemic in Bucharest. In the last decades an increasing number of WND cases was observed in equines and humans [1] with a consequent increase in the official notifications. Phylogenetic analyses revealed that all European WNV lineage 1 and 2 strains are derived from a limited number of independent introductions, most likely from Africa, followed by local spread and evolution [2].

The transmission, epidemiology and geographic distribution of WNV are the result of the interaction between a wide range of climate and environmental factors affecting, to a different extent, both host and vector populations (Chevalier n°5). Preliminary attempts to investigate these complex interactions have been reported since the 1950s in Egypt [3], and further explored identifying several factors able to influence the disease spread and persistence [4]. The availability of remote sensed data have made data collection easier and faster so to promote the development of modelling and spatial analyses at large scale [5,6]. Among the most relevant variables, temperature and vegetation indices are often used as covariate and found to be significant in the transmission of the virus [5,7–10]. However, despite the availability of such datasets, their visualization and integration into accessible information systems have been only recently developed [11].

The need of global systems for animal disease surveillance is gaining considerable attention and, at the same time, the increased power of technology, together with advanced informatics tools, are facilitating the collection and sharing of relevant epidemiological data [12–14]. The effectiveness of any surveillance system relies on rapid and complete data collection and dissemination to provide decision makers and stakeholders with real time information to support the prompt application of control and preventive measures.

The Italian National Reference Center for Foreign Animal Disease (CESME) and the Italian National Reference Center for Epidemiology (COVEPI) developed a web based application, called Disease Monitoring Dashboard (DMD) in which data on WNV occurrence in Europe and the Mediterranean basin are collected from multiple sources and displayed on interactive maps. Climatic and environmental data are integrated into the DMD with genomic and epidemiological details for the recent WNV outbreaks and basic epidemiological tools have been developed. This paper describes the DMD system, the tools available for the preliminary analysis on climatic and environmental factors and the other interactive tools for epidemiological analysis.

## Methods

### Data collection

**Fig 1:**
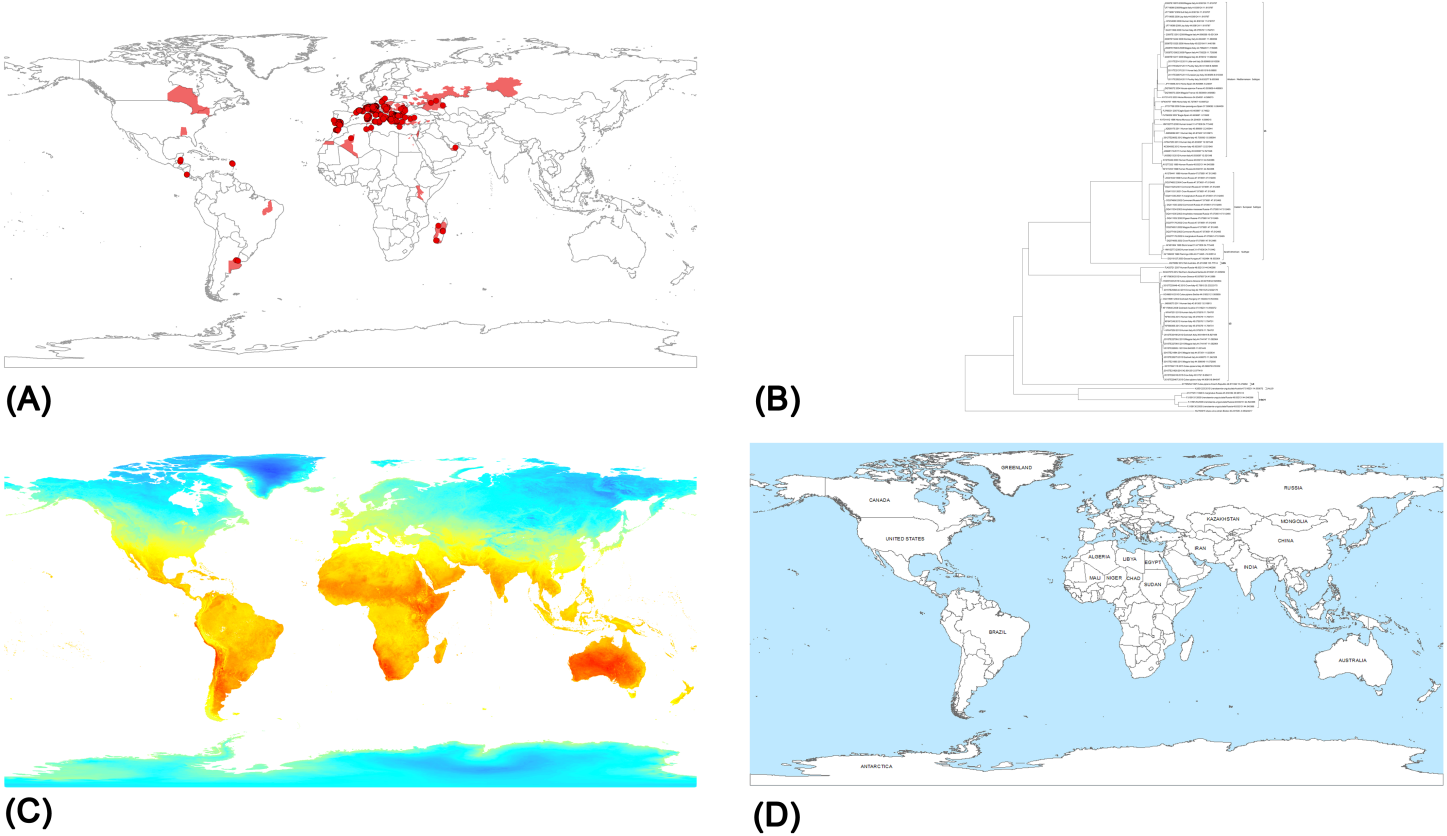
**Datasets displayed by the web GIS**–**(A)** WNV distribution and outbreaks since 1994 (points for outbreaks and polygons for disease distributions), **(B)** Phylogenetic tree of selected WNV strains, **(C)** MODIS Day Land Surface Temperature (DLST) January 2011, and **(D)** Geographical distribution of the Countries. (The MODIS image derives from open data downloaded from NASA EOSDIS Land Processes DAAC, USGS Earth Resources Observation and Science (EROS) Center at http://eros.usgs.gov/# (https://lpdaac.usgs.gov). The shapefile related to admistrative units of the countries is downloaded from Natural Earth at http://www.naturalearthdata.com/).

The DMD database integrates disease information, genomic sequences and climatic-environmental data (Fig 1), collected from different sources:

1. Official epidemiological information are retrieved from the World Animal Health Information System (WAHIS—OIE), World Health Organization (WHO), Animal Disease Notification System (ADNS–European Commission), European Centre for Disease Prevention and Control (ECDC) and from the Italian integrated national surveillance plan [12–15]; not official sources are mainly scientific publications or reports (ProMed, Eurosurveillance, etc). When available the details collected for each outbreak include country/region/province or point (latitude and longitude) of occurrence, outbreak code, number of cases, susceptible, destroyed, slaughtered and dead animals, date of occurrence and confirmation of the event, species, virus lineage and data source. The disease data aggregation depends on the geographic unit available.
2. Whole genome sequences and metadata of WNV strains are retrieved from public databases (e.g. strains from Italy, Austria, Spain, Serbia, Hungary, Czech Republic, Greece, Morocco, Israel and Russia) or generated in the framework of the Italian surveillance activities [16]. In particular West Nile virus fasta sequences were automatically downloaded from GeneBank [17] by using the NCBI eutils package [18] and the NCBI taxonomic ID as query. Incomplete genome sequences were filtered out. Metadata such as host, country of isolation and date of collection, derived from their corresponding genebank files, were merged to the sequences fasta header by an in-house developed python script. Latitude and longitude were added when available. Conversely, the centroid of the country/region/province of isolation was assigned.
  Sequences were aligned with Mafft aligner [19] using the "—auto" parameter to balance speed and accuracy on the basis of the input file size. Returned alignment was used as input to build a Neighbor Joining Tree by using the NJTree software [20] with default parameters and 1000 bootstrap replicates. The Usutu Biotec strain (KU760915) was used as out-group. Trees were visualized to draw figures by Figtree [21].
  A collection of python scripts to download sequences and their metadata, merging them, running the alignment and the tree building and finally reformatting tree for the web, were in-house developed.
3. Environmental and climatic data are gathered from the NASA Land Processes Distributed Active Archive Center (LP DAAC) [22,23]: MODIS Land Surface Temperature & Emissivity (MOD11C3.006) and MODIS Vegetation Indices (MOD13C2.006) at the 5 km spatial resolution and one month temporal resolution. The system manages four cubes of monthly images at world coverage:
  - Day Land Surface Temperature (DLST)
  - Night Land Surface Temperature (NLST)
  - Normalized Difference Vegetation Index (NDVI)
  - Enhanced Vegetation Index (EVI).
  MODIS data are obtained downloading the HDF (Hierarchical Data Format) compressed files. A first automated pre-processing python procedure extracts the DLST and NLST levels from MOD11C3.006 product and the NDVI and EVI levels from MOD13C2.006. Raster datasets are added to a data structure called ‘Mosaic Dataset’, organized in time series mode. A second automated python procedure provides the link between disease outbreaks and vegetation indices and temperature values, using their geographic location.
4. Tiled Map Services (TMS) by ESRI: World Topographic Map, World Street Map, Light Gray Canvas Map and World Imagery.

Epidemiological data are updated in near real-time mode while environmental and climatic data are updated monthly (the most recent updated images refer to the month before the upper bound time window). Availability of novel genomic sequences is verified on a regular base and downloaded from GeneBank as previously described.

**Fig 2:**
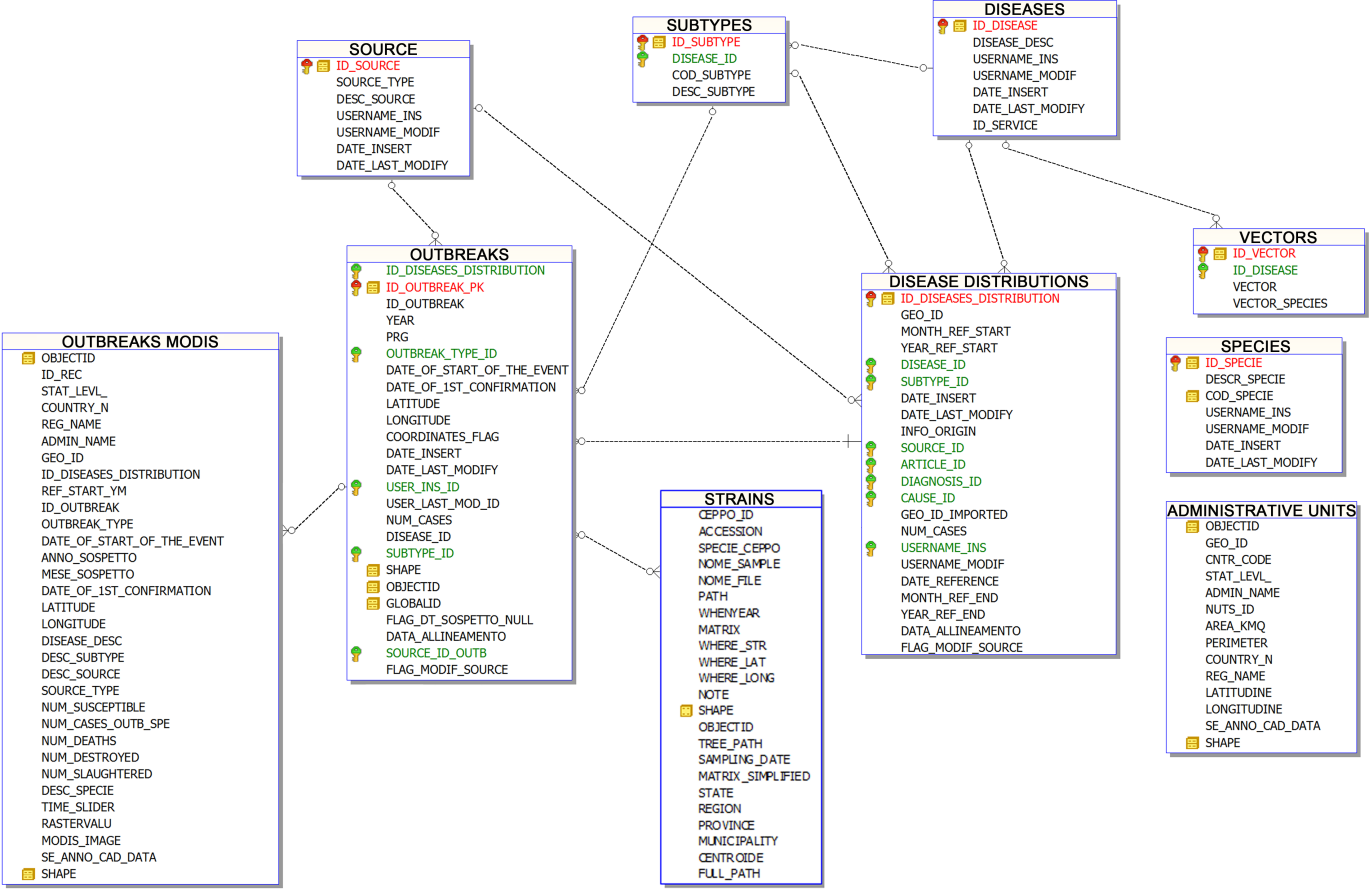
Database structure and table relationship. The DMD database contains a set of support tables to facilitate the database updating (i.e. the decoding tables of diseases, species, diagnosis and vectors), and tables containing epidemiological information and environmental and climatic data.

The Geographic Reference System is GCS WGS84. Database structure and table relationship is showed in Fig 2.

### System technology and architecture

**Fig 3:**
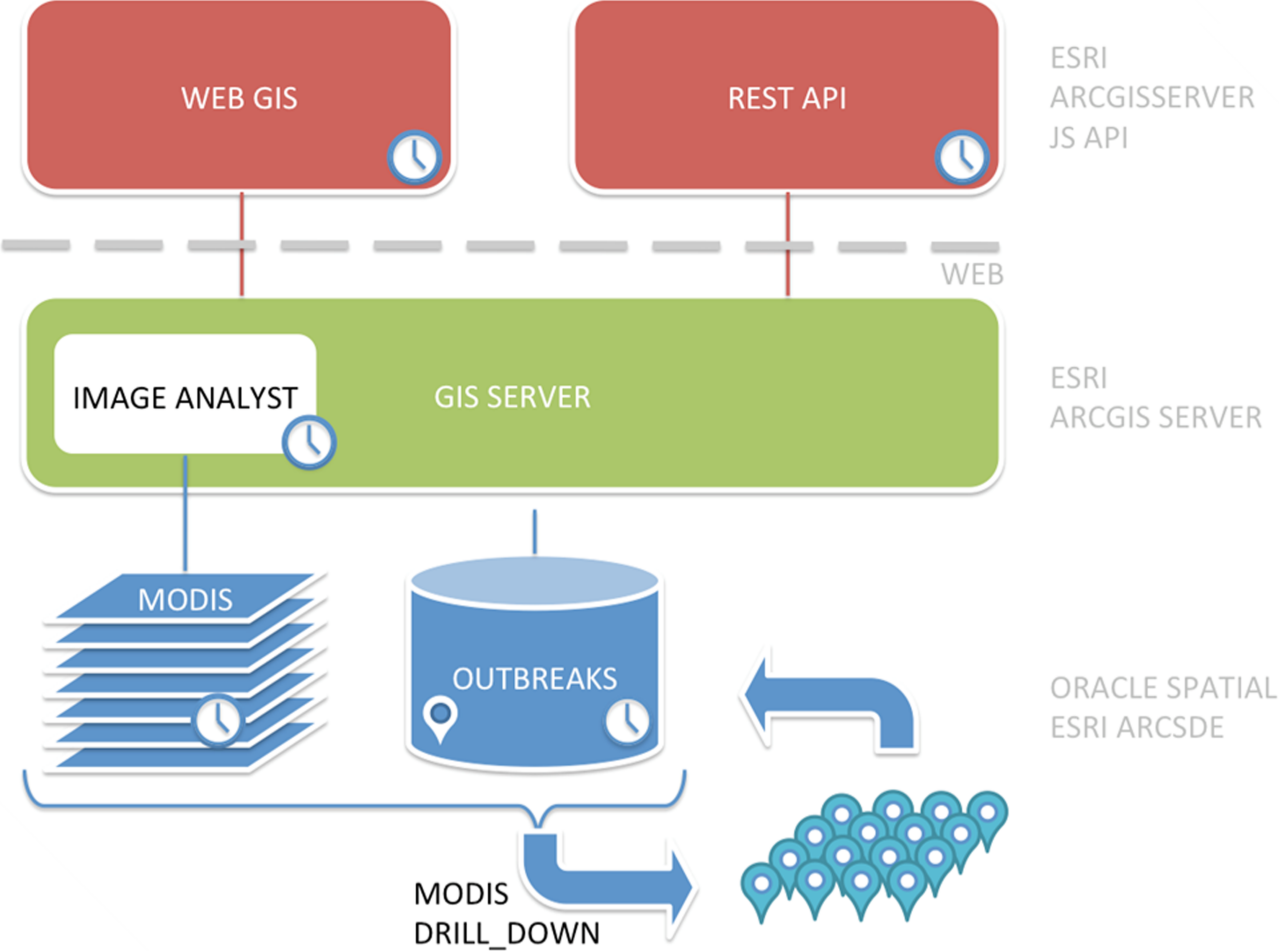
DMD system architecture.

The DMD system architecture is composed by three application levels (Fig 3):

- a data level composed by the spatial database (outbreaks points and disease distribution polygons, strains) and the multi temporal MODIS image repository;
- an application server level which includes the GIS engine and several ReST geowebservices;
- a presentation level that includes a JavaScript client dashboard for the data exploration, consuming the geowebservices provided by the server.

The infrastructure of the Geographic Information System (GIS) is composed by Oracle 10g DataBase Management System and ArcGIS Server Enterprise, ArcGIS for Desktop (ESRI® Inc., Redlands, CA, USA). ArcGIS Server spread the ArcGIS Desktop project and its layers in the form of ReST web services.

The web mapping application is developed with the ArcGIS API for JavaScript and Phylocanvas library (dedicated to the visualization of the phylogenetic trees) and allows interaction with the data exposed through ReST services. Finally, the Twitter Bootstrap framework has been used to realize the front-end of the portal, giving a homogeneous style to the different pages.

### DMD portal

The DMD portal home page contains two main sections: Database and Web GIS.

Database section allows authorized users to query and manage epidemiological information stored in the database; the Web GIS section is freely accessible and provides the spatio-temporal consultation of epidemiological, environmental and climatic data through an interactive and easy to use application. Two sub-sections are available: Disease and PhyloWN.

The **Disease** section is the core application and displays all the collected information managed through different tools:

- **Filters**—several query can be run to choose disease related attributes (species, lineage, etc.), time of occurrence, location;
- **Outbreaks graph**—it shows the epidemiological curve in the chosen period. A time slider (date from–date to) allows a dynamic view of the epi-curve;
- **Environment**—two different environmental analyses can be run in relation to a selected outbreak, the first one reporting the six month values of DLST, NLST, NDVI, EVI, preceding the upper limit of the time slider; the second one reporting the six month values of DLST, NLST, NDVI, EVI preceding the start date of the outbreak. The outputs of the analysis are either listed or showed in a graph;
- **Map**—different layers can be turned on and off as background map and a legend is provided.

The **PhyloWN** section displays map location and the phylogenetic tree of the selected strains.

## Results

**Fig 4:**
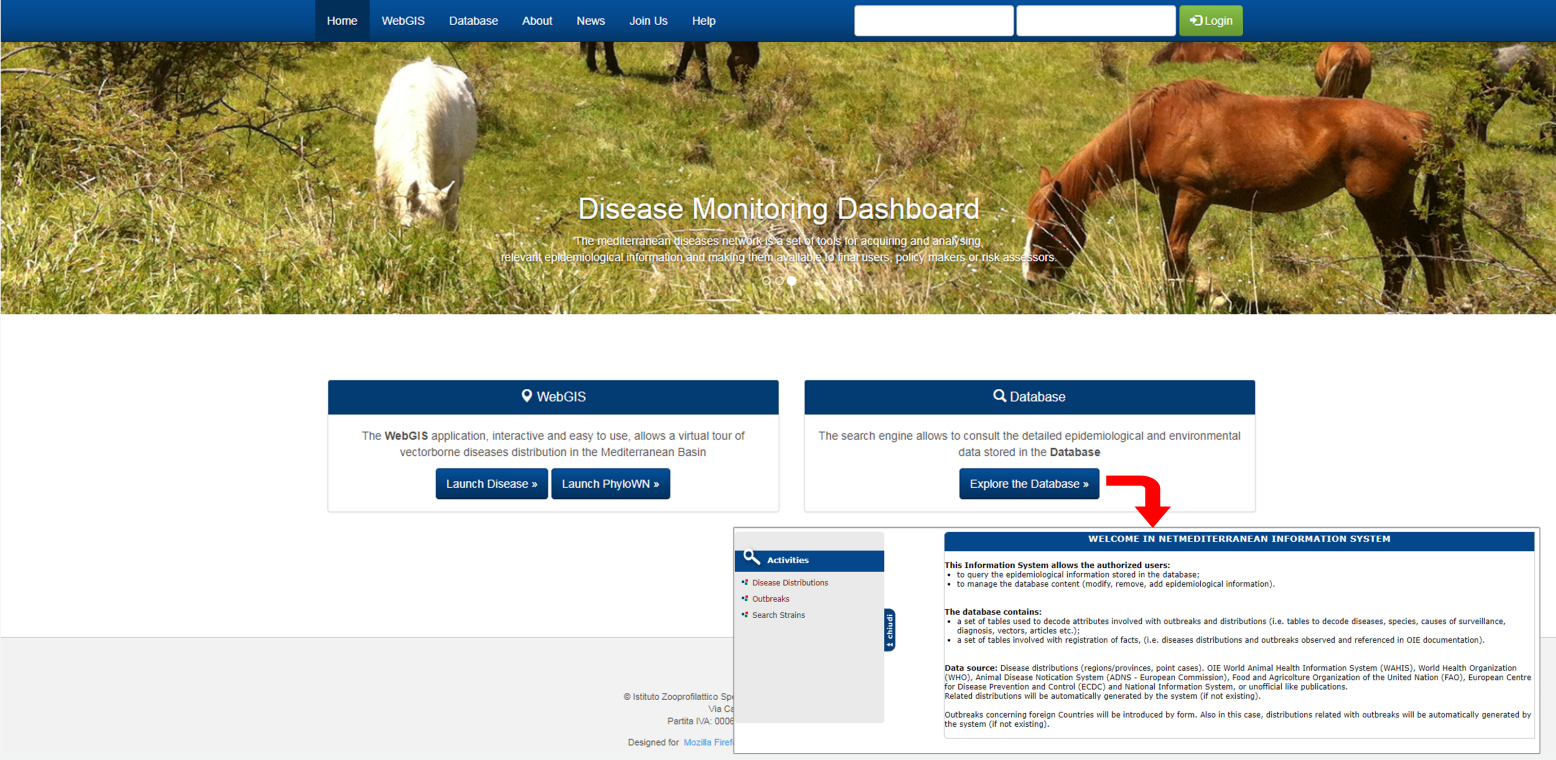
Homepage of the DMD portal. The ‘Database' section allow the user to access database tables to consult (by system’s public access) or add, edit, and delete epidemiological data (by system’s authorized access).

The DMD web application is accessible through a generic web browser at https://netmed.izs.it/networkMediterraneo/, Fig 4 shows the two main sections: Database and Web GIS sections.

### Database

Database section can accessed in public and authorized form: all users can search and display data in table format, authorized users can add, edit and remove the epidemiological information. All the edit actions are tracked in the database to ensure traceability.

To date, the database includes WNV outbreaks starting from 1994 involving human and veterinary cases (Table 1). The disease distribution includes 307 different administrative units around the world (depending on data availability). The database is fed both online by ad-hoc forms and offline (MODIS data), using automatic procedures in both cases. WND Italian data are automatically uploaded from the National Surveillance System. The MODIS database currently stores 593 images, collected since 2001 for climatic data and 2010 for environmental data respectively at global level. The PhyloWN tree has been currently developed using these 95 WNV whole genome sequences (Table 1) while the Usutu Biotech strain (JX276662) has been used to root the tree.

**Table 1:** Number of WNV outbreaks and strains stored in the DMD database since 1994.

Species	Cases	Strains
Humans	229	24
Arthropods	332	15
Birds	295	47
Horses	1079	8
***Total***	***1935***	94*

*The total number of strains doesn’t sum up to 95 because the Kunjin strain (JX276662) has been sequenced from the strain CH16532 stored in the “KV Master Virus Bank” without specie specification.

### Web GIS

**Fig 5:**
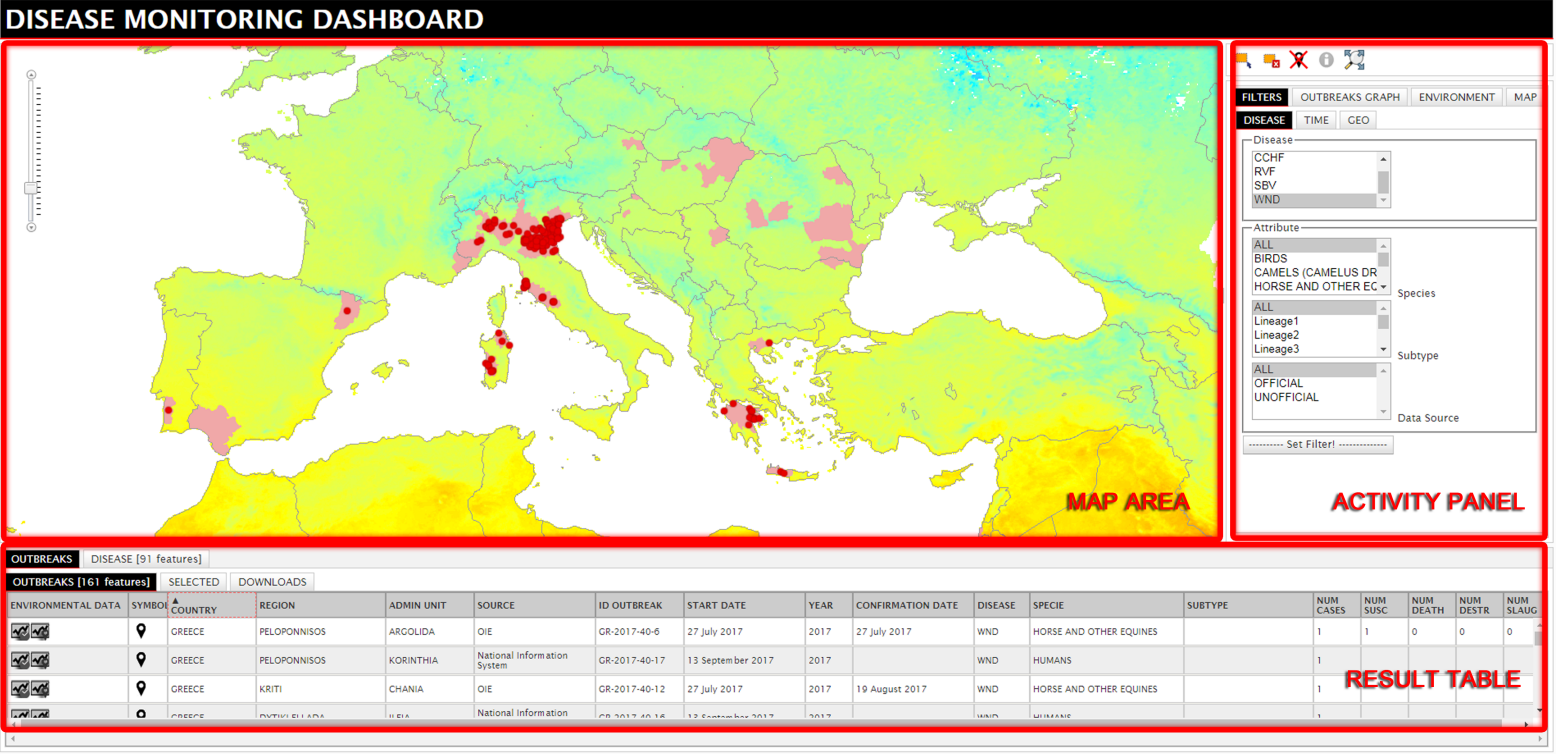
Disease section interface. (The MODIS image derives from open data downloaded from NASA EOSDIS Land Processes DAAC, USGS Earth Resources Observation and Science (EROS) Center at http://eros.usgs.gov/# (https://lpdaac.usgs.gov)).

The Disease section interface is structured in three areas: ‘Map area’, ‘Activity panel’ and ‘Result table’. The opening layers displayed on the map reports WNV distribution from 1994 up to now and the World Topographic Map as basemap (Fig 5).

The set of filters in the activity panel allows the user to customize the dataset and visualize the required information in map and table format. The available filters are divided in ‘DISEASE’, ‘TIME’ and ‘GEO’ tabs. ‘DISEASE’ filter specifies the disease selection (currently available data on WND), host species, subtype (lineage) and data source (official / unofficial); ‘TIME’ filter defines the time window for data enquire; ‘GEO’ filter identifies the geographic area of interest.

Map and table are interactively connected so that a selection of a record in the table highlights the point location on the map and vice versa. Outbreaks can also be selected through the ‘selection’ or ‘identifier’ tools in the activity panel. In the ‘DOWNLOADS’ tab the selected outbreaks can be downloaded in excel spreadsheet format (with a limit of 1000 selected items) together with the environmental and climatic values of the specified year.

**Fig 6:**
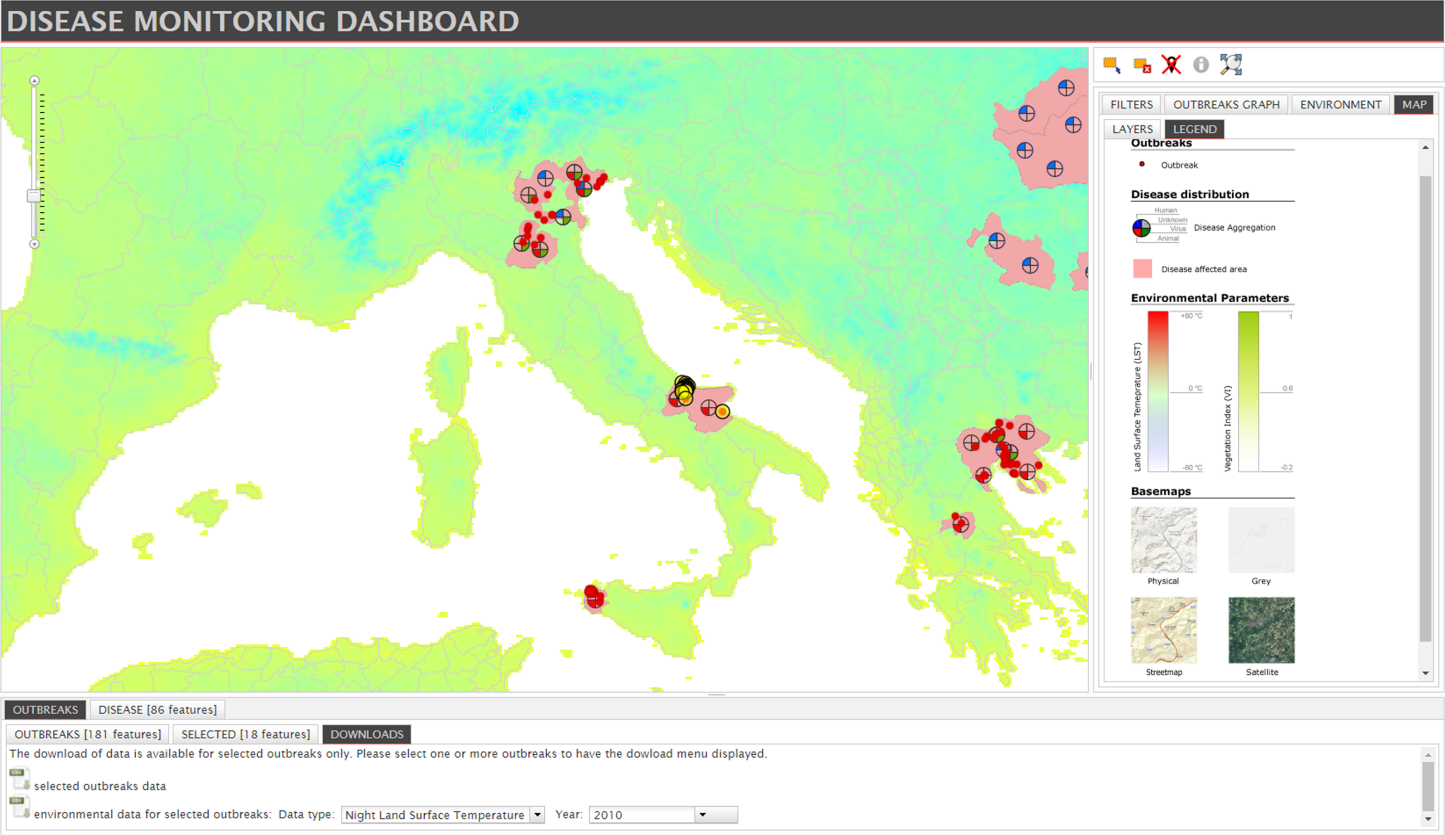
‘DOWNLOAD’ tab to download the list of selected outbreaks and the related environmental and climatic data as excel spreadsheet format. ‘LEGEND’ panel to properly interpret the symbols and colours in the disease map.

The ‘LEGEND’ panel shows the symbols and colours used in the map; the pie chart is the symbol linked to the ‘Disease distribution’ layer and represents the multiple species involved at Nuts 3 level: mosquitoes and birds (virus group), humans, animals or unknown (not reported species) in the affected administrative unit (Fig 6).

**Fig 7:**
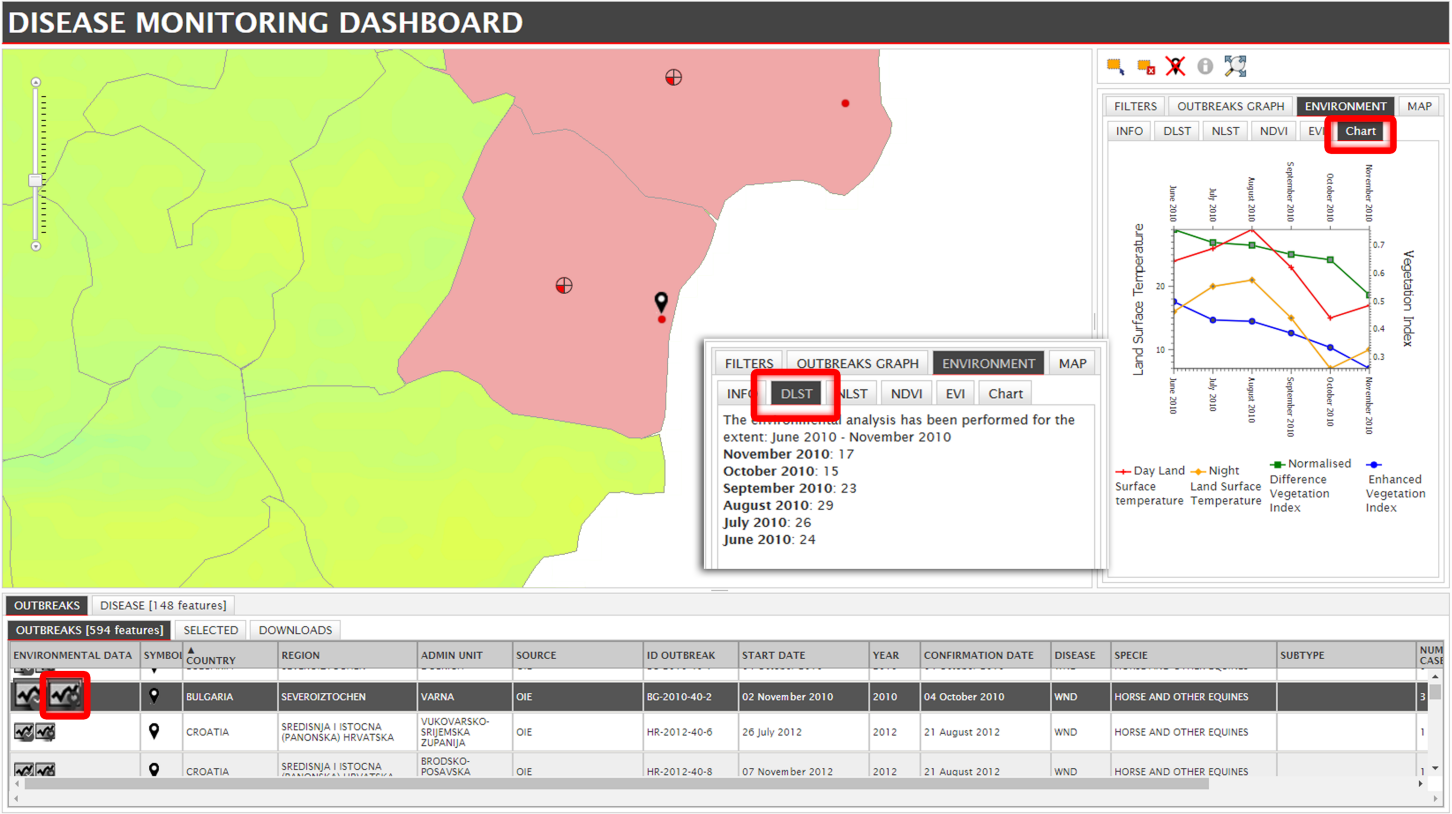
Preliminary environmental and climatic analysis. Values for the 6 months preceding the start date of the outbreak are displayed in tab (DLST) and graph format (Chart tab). (The MODIS image derives from open data downloaded from NASA EOSDIS Land Processes DAAC, USGS Earth Resources Observation and Science (EROS) Center at http://eros.usgs.gov/# (https://lpdaac.usgs.gov)).

The Disease section integrates epidemiological, environmental and climatic data, by combining information from multiple sources. A preliminary environmental and climatic analysis can be run for each outbreak location, producing a graph reporting values of temperatures and vegetation indices in the six months preceding the start date of the outbreak (Fig 7).

**Fig 8:**
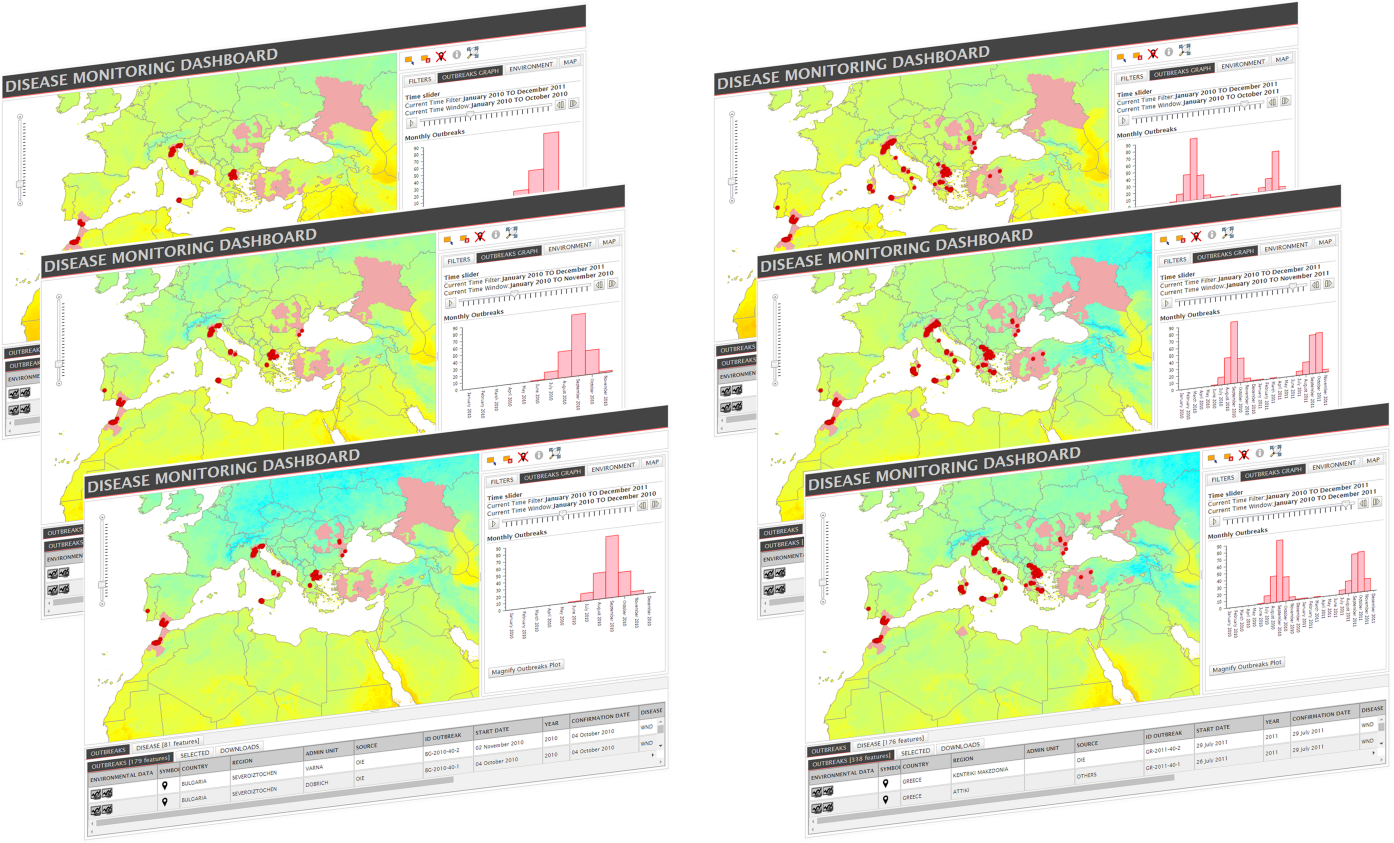
Time slider for the time windows October-December 2010 and October-December 2011. MODIS NLST images in the selected period change from month to month. (The MODIS images derive from open data downloaded from NASA EOSDIS Land Processes DAAC, USGS Earth Resources Observation and Science (EROS) Center at http://eros.usgs.gov/# (https://lpdaac.usgs.gov)).

A visualization in space and time is available through a time slider tool which animates outbreaks, disease distribution data and MODIS images on the map and the epidemic curve graph (Fig 8).

**Fig 9:**
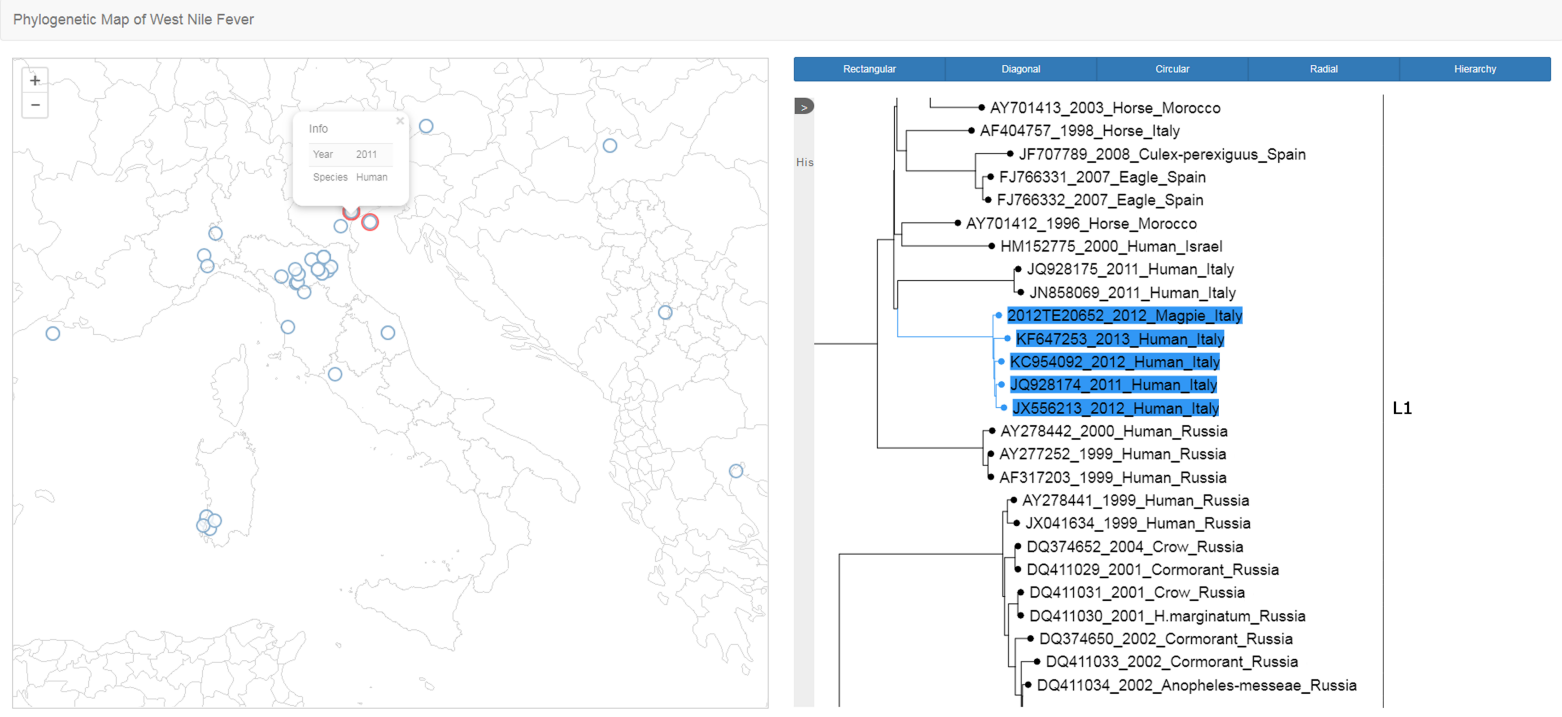
PhyloWN section interface for 95 selected West Nile strains. Left side: map with the selected strains; right, the phylogenetic tree (rectangular mode). (The shapefile related to admistrative units of the countries is downloaded from Natural Earth at http://www.naturalearthdata.com/).

The PhyloWN section displays map location and phylogenetic clustering tree of the selected strains. Strains are localized according to the geographic coordinates, when available, or the centroid of the administrative unit of reference. Map and tree interact each other: by selecting a point on the map the corresponding strain is highlighted in the tree while selection of a node in the tree highlights all the strains belonging to the branch on the map (Fig 9). The WNV complete sequences grouped according to the genetic lineages and, within each lineage, clustering reflects the geographic origin of the circulating strains and/or the year of the viral circulation. The most broadly represented clusters are those belonging to lineage 1, with the Western Mediterranean, the Eastern European and the Israeli-American subtypes and lineage 2, which includes the most recent circulating strains in EU countries. The remaining clusters, consisting of one or few sequences represent the prototype of proposed novel lineages as the WNV-Uu-LN-AT-2013 strain from Austria (KJ831223) prototype of lineage 9, the Rabensburg strain (lineage 3) and the group of WNV sequences isolated from *Ur*. *unguiculata* mosquitoes and *Dermacentor marginatus* ticks in Russia (lineage 4).

The phylogenetic tree has also different styles of representation: rectangular, diagonal, circular, radial and hierarchy.

## Discussion

In the last decade, many systems have been developed in animal and public health sectors for collection and dissemination of disease information worldwide. The establishment of disease databases provides structured scientific information and allows flexible incorporation of data coming from multiple sources. Long-term massive heterogeneous data can be nowadays managed, analysed and visualised by regular updating with well-defined user interfaces.

To this purpose, OIE launched in 2005 the World Animal Health Information System, known by its acronym WAHIS [13], which manages the notification of animal health disease events provided by Member Countries in their national territory. The WAHIS Interface provides access to all data held within the OIE Information System. However the WAHIS, as other available web systems (e.g. Animal Disease Notification System–EC [12], Center for Disease Control and Prevention–CDC [24]) is currently lacking of an interactive and dynamic mapping tool that would empower the disease spread visualization.

Web GIS tools have been used to overcome such limits, and publicly accessible GIS for displaying, sharing and analysing spatial data have been recently developed [11,25,26]. Such systems are fundamental tools for collecting relevant epidemiological information and making them available to final users, policy makers or risk assessors.

In Europe, Member States (MS) should monitor WNV activity, if warranted by the epidemiological situation (Directive 2003/99/EC on the monitoring of zoonosis and zoonotic agents) and, since 2012, MS agreed to report WND cases in animals. Surveillance of WNV circulation is challenging since it requires an interdisciplinary approach with the integration of entomological, veterinary and human surveillance systems; all these systems provide a complex amount of data, fundamental for estimating the public health risk associated with WNV, and for the effective and timely control of the disease in humans [15].

In this framework the DMD system facilitates near real-time managing, sharing, visualization and analysis of epidemiological data related to WND in the Mediterranean basin through the integrated use of dynamic maps, phylogenetic trees, graphs and tables.

Being able to associate epidemiological data, environmental factors together with the genetic mapping of the circulating strains provides a tremendous advantage in terms of real-time monitoring for the introduction of new WNV strains or the selection of novel viral variants with modified/improved pathogenicity in affected areas [27]. Unfortunately the limiting factor is represented by the scarce number of genomic sequences of the WNV and their limited geographical representativeness in the affected area of the Mediterranean region but such limitation will be likely overcome in the very next future due to the improvement in sequencing technologies.

For this purpose the system compiles multiple datasets through user-friendly web tools; it integrates entomological, veterinary and human data, molecular information on pathogens and environmental and climatic data and provides spatio-temporal tools for preliminary epidemiological analysis. The system will benefit of accurate data on outbreak locations that are often difficult to obtain although global surveillance and data availability are improving.

In the next future, other pathogens and diseases will be included in the system so as to make more extensive and accurate information available to risk assessors and decision makers in order to develop strategies for integrated prevention and control measures of animal and human diseases.
